# The genome sequence of *Tachina fera *Linnaeus, 1761, a tachinid fly

**DOI:** 10.12688/wellcomeopenres.17760.1

**Published:** 2022-03-21

**Authors:** University of Oxford and Wytham Woods Genome Acquisition Lab, Darwin Tree of Life Barcoding collective, Wellcome Sanger Institute Tree of Life programme, Wellcome Sanger Institute Scientific Operations: DNA Pipelines collective, Tree of Life Core Informatics collective, Will Nash, Darwin Tree of Life Consortium

**Affiliations:** 1 Earlham Institute, Norwich, UK

**Keywords:** Tachina fera, genome sequence, chromosomal, Diptera

## Abstract

We present a genome assembly from an individual female *Tachina fera* (Arthropoda; Insecta; Diptera; Tachinidae). The genome sequence is 752 megabases in span. The majority of the assembly (99.98%) is scaffolded into 6 chromosomal pseudomolecules, with the X sex chromosome assembled. The complete mitochondrial genome was also assembled and is 17.4 kilobases in length.

## Species taxonomy

Eukaryota; Metazoa; Ecdysozoa; Arthropoda; Hexapoda; Insecta; Pterygota; Neoptera; Endopterygota; Diptera; Brachycera; Muscomorpha; Oestroidea; Tachinidae; Tachininae; Tachinini; Tachina; *Tachina fera* Linnaeus, 1761 (NCBI:txid631328).

## Background

*Tachina fera* (Linnaeus, 1761) is one of the most striking flies commonly encountered in the UK countryside. With adults ranging between 9 and 14 mm in length, it is an easily noticeable fly. Spiky bristles, characteristic of the Tachinidae, adorn a chestnut abdomen with a dark central stripe. *Tachina fera* is abundant across Europe, North Africa and Asia ( Tschorsnig & Herting, 1994). In the UK, *T. fera* is bivoltine, with adults in flight from May to June, and from July to September ( Belshaw, 1993). Adults feed at a range of flowers throughout the landscape. *Tachina fera* has mainly been recorded emerging from Noctuid moth caterpillars ( Belshaw, 1993). The method of parasitism utilised by *T. fera* is notable as the egg is not placed into the host by the mother but laid pre-incubated onto leaves close to it. The larva, once hatched, will make its own way to the host, stimulated by vibration ( Belshaw, 1993; Stireman *et al.,* 2006). The parasitic nature of Tachinid species such as *T. fera* mean they are important, but underappreciated, regulators of insect herbivory in our ecosystem ( Stireman *et al.,* 2006), as well as playing important roles in pollination (e.g. Martel *et al.,* 2021). The chromosome-level genome assembly presented here is, to our knowledge, the first high-quality resource developed for a Tachinid and represents a key step in understanding the complex ecology of these beautiful and spiky flies.

## Genome sequence report

**Figure 1.**
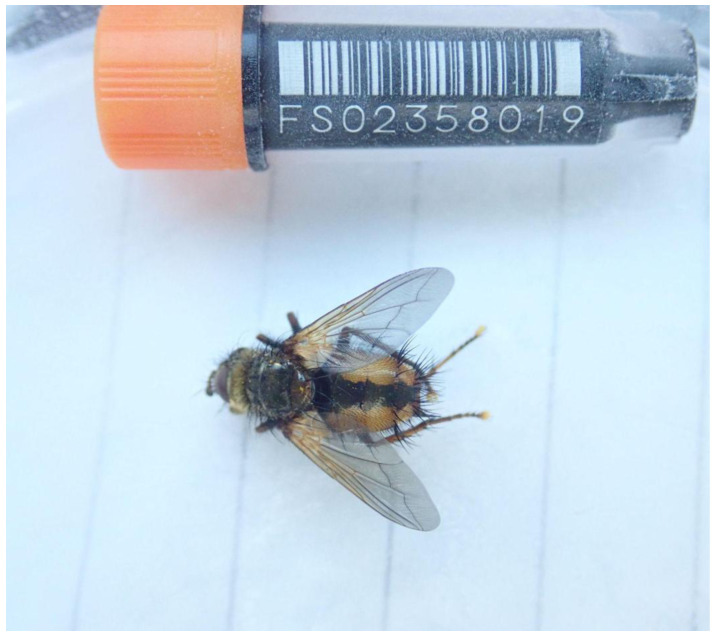
Image of the *Tachina fera* specimen taken during preservation and processing.

The genome was sequenced from a single female *T. fera* collected from Wytham Woods, Oxfordshire (Biological vice-county: Berkshire), UK (latitude 51.770, longitude -1.338) ( Figure 1). A total of 41-fold coverage in Pacific Biosciences single-molecule HiFi long reads and 46-fold coverage in 10X Genomics read clouds were generated. Primary assembly contigs were scaffolded with chromosome conformation Hi-C data. Manual assembly curation corrected 246 missing/misjoins and removed 60 haplotypic duplications, reducing the assembly size by 1.88% and the scaffold number by 94.81%, and increasing the scaffold N50 by 120.51%.

**Figure 2.**
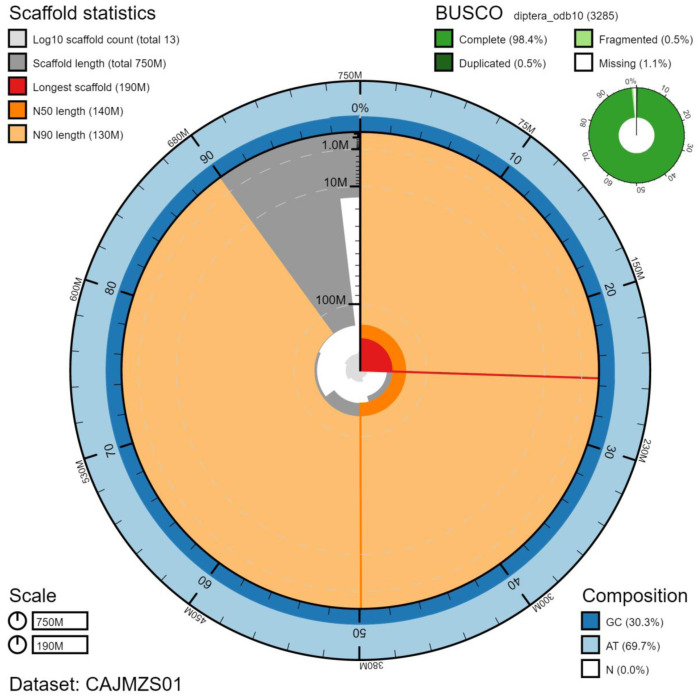
Genome assembly of *Tachina fera*, idTacFera2.1: metrics. The BlobToolKit Snailplot shows N50 metrics and BUSCO gene completeness. The main plot is divided into 1,000 size-ordered bins around the circumference with each bin representing 0.1% of the 751,737,434 bp assembly. The distribution of chromosome lengths is shown in dark grey with the plot radius scaled to the longest chromosome present in the assembly (191,818,649 bp, shown in red). Orange and pale-orange arcs show the N50 and N90 chromosome lengths (141,997,299 and 125,182,871 bp), respectively. The pale grey spiral shows the cumulative chromosome count on a log scale with white scale lines showing successive orders of magnitude. The blue and pale-blue area around the outside of the plot shows the distribution of GC, AT and N percentages in the same bins as the inner plot. A summary of complete, fragmented, duplicated and missing BUSCO genes in the diptera_odb10 set is shown in the top right. An interactive version of this figure is available at https://blobtoolkit.genomehubs.org/view/idTacFera2.1/dataset/CAJMZS01/snail.

The final assembly has a total length of 752 Mb in 12 sequence scaffolds with a scaffold N50 of 142 Mb ( Table 1). The majority, 99.98%, of the assembly sequence was assigned to 6 chromosomal-level scaffolds, representing 5 autosomes (numbered by sequence length), and the X sex chromosome ( Figure 2– Figure 5; Table 2). The order and orientation of contigs within the centromere of chromosome 2 are not known. Lots of apparent haplotypic duplication was excised from this region owing to a divergent Hi-C pattern and seeming low coverage (which was somewhat ambiguous due to read coverage levels in this repetitive region).

**Table 1.** Genome data for *Tachina fera*, idTacFera2.1.

*Project accession data*	
Assembly identifier	idTacFera2.1
Species	*Tachina fera*
Specimen	idTacFera2
NCBI taxonomy ID	631328
BioProject	PRJEB42946
BioSample ID	SAMEA7520333
Isolate information	Female, thorax/abdomen (idTacFera2, genome assembly, Hi-C); unknown sex, abdomen (idTacFera1, RNA-Seq)
*Raw data accessions*	
PacificBiosciences SEQUEL II	ERR6608654
10X Genomics Illumina	ERR6054375-ERR6054378
Hi-C Illumina	ERR6054379-ERR6054381
PolyA RNA-Seq Illumina	ERR6054382
*Genome assembly*	
Assembly accession	GCA_905220375.1
*Accession of alternate haplotype*	GCA_905220395.1
Span (Mb)	752
Number of contigs	322
Contig N50 length (Mb)	16.2
Number of scaffolds	12
Scaffold N50 length (Mb)	142
Longest scaffold (Mb)	192
BUSCO * genome score	C:98.4%[S:97.9%,D:0.5%], F:0.5%,M:1.1%,n:3285

*BUSCO scores based on the diptera_odb10 BUSCO set using v5.1.2. C= complete [S= single copy, D=duplicated], F=fragmented, M=missing, n=number of orthologues in comparison. A full set of BUSCO scores is available at https://blobtoolkit.genomehubs.org/view/idTacFera2.1/dataset/CAJMZS01/busco.

**Figure 3.**
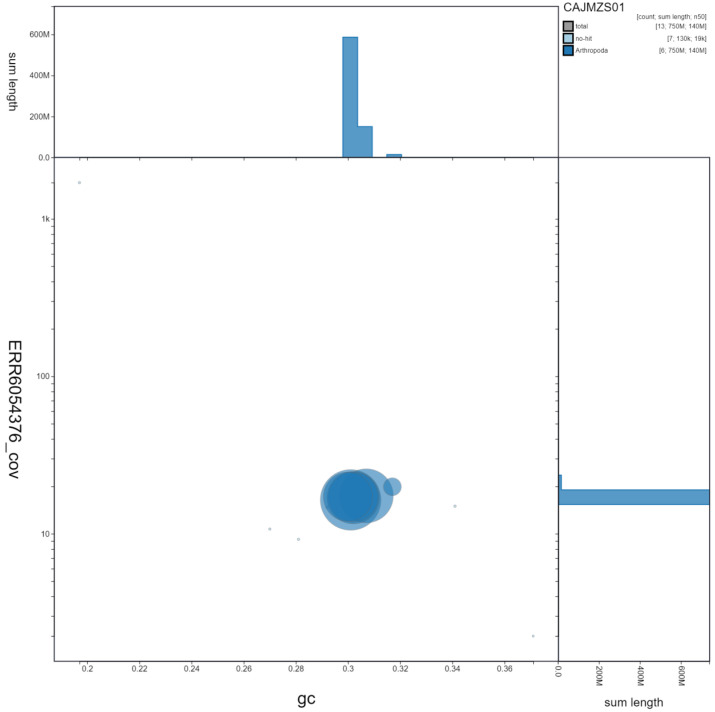
Genome assembly of *Tachina fera*, idTacFera2.1: GC coverage. BlobToolKit GC-coverage plot. Scaffolds are coloured by phylum. Circles are sized in proportion to scaffold length. Histograms show the distribution of scaffold length sum along each axis. An interactive version of this figure is available at https://blobtoolkit.genomehubs.org/view/idTacFera2.1/dataset/CAJMZS01/blob.

**Figure 4.**
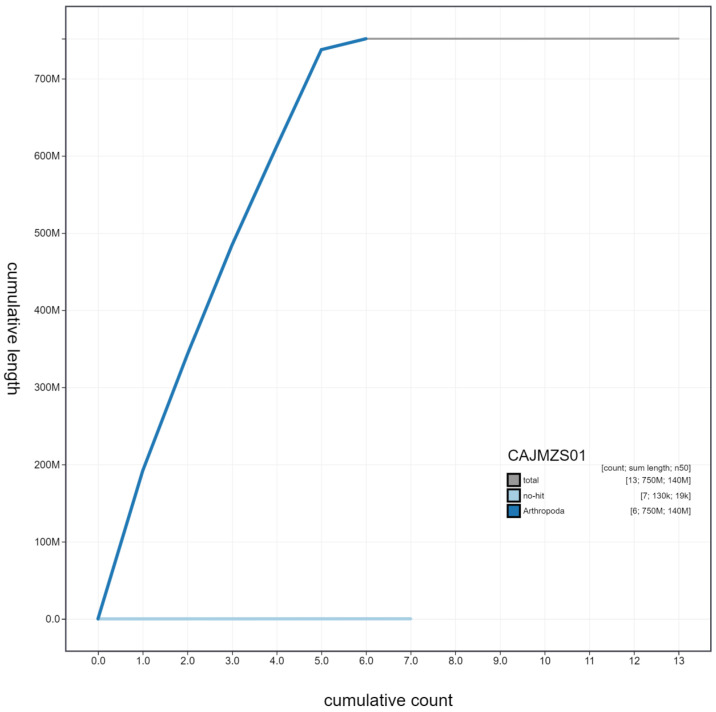
Genome assembly of *Tachina fera*, idTacFera2.1: cumulative sequence. BlobToolKit cumulative sequence plot. The grey line shows cumulative length for all scaffolds. Coloured lines show cumulative lengths of scaffolds assigned to each phylum using the buscogenes taxrule. An interactive version of this figure is available at https://blobtoolkit.genomehubs.org/view/idTacFera2.1/dataset/CAJMZS01/cumulative.

**Figure 5.**
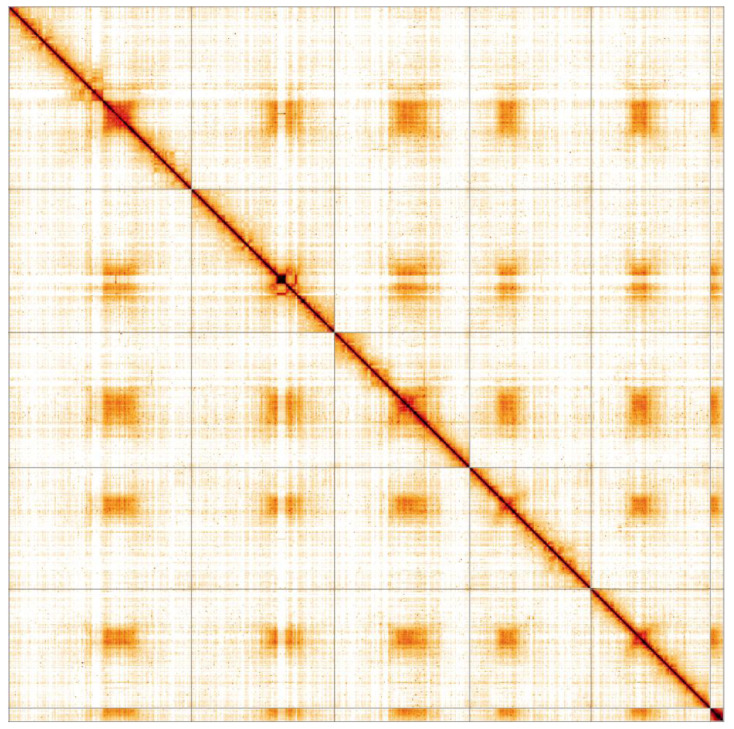
Genome assembly of *Tachina fera*, idTacFera2.1: Hi-C contact map. Hi-C contact map of the idTacFera2.1 assembly, visualised in HiGlass. Chromosomes are arranged in size order from left to right and top to bottom.

**Table 2.** Chromosomal pseudomolecules in the genome assembly of *Tachina fera*, idTacFera2.1.

INSDC accession	Chromosome	Size (Mb)	GC%
LR999963.1	1	191.82	30.1
LR999964.1	2	150.60	30.7
LR999965.1	3	142.00	30.2
LR999966.1	4	127.83	30.2
LR999967.1	5	125.18	30.0
LR999968.1	X	14.18	31.7
LR999969.1	MT	0.02	19.9
-	Unplaced	0.11	30.2

The assembly has a BUSCO v5.1.2 ( Manni *et al.,* 2021) completeness of 98.4% (single 97.9%, duplicated 0.5%) using the diptera_odb10 reference set (n=3285). While not fully phased, the assembly deposited is of one haplotype. Contigs corresponding to the second haplotype have also been deposited.

## Methods

### Sample acquisition and DNA extraction

One female *T. fera* sample (idTacFera2), and a second sample of unknown sex (idTacFera1) was collected from Wytham Woods, Oxfordshire (Biological vice-county: Berkshire), UK (latitude 51.770, longitude -1.338) by Liam Crowley, University of Oxford, on 15 June 2020. The specimen was caught in woodland with a net, identified by the same individual, snap-frozen on dry ice and stored using a CoolRack.

DNA was extracted from the head/thorax of idTacFera2 at the Wellcome Sanger Institute (WSI) Scientific Operations core using the Qiagen MagAttract HMW DNA kit, according to the manufacturer’s instructions. RNA (from the abdomen of idTacFera1) was extracted in the Tree of Life Laboratory at the WSI using TRIzol, according to the manufacturer’s instructions. RNA was then eluted in 50 μl RNAse-free water and its concentration RNA assessed using a Nanodrop spectrophotometer and Qubit Fluorometer using the Qubit RNA Broad-Range (BR) Assay kit. Analysis of the integrity of the RNA was done using Agilent RNA 6000 Pico Kit and Eukaryotic Total RNA assay.

### Sequencing

Pacific Biosciences HiFi circular consensus and 10X Genomics Chromium read cloud sequencing libraries were constructed according to the manufacturers’ instructions. Sequencing was performed by the Scientific Operations core at the Wellcome Sanger Institute on Pacific Biosciences SEQUEL II (HiFi), Illumina HiSeq X (10X) and Illumina HiSeq 4000 (RNA-Seq) instruments. Hi-C data were generated in the Tree of Life laboratory from remaining head/thorax tissue of idTacFera2 using the Arima v2 kit and sequenced on a HiSeq X instrument.

### Genome assembly

Assembly was carried out with Hifiasm ( Cheng *et al.,* 2021); haplotypic duplication was identified and removed with purge_dups ( Guan *et al.,* 2020). One round of polishing was performed by aligning 10X Genomics read data to the assembly with longranger align, calling variants with freebayes ( Garrison & Marth, 2012). The assembly was then scaffolded with Hi-C data ( Rao *et al.,* 2014) using SALSA2 ( Ghurye *et al.,* 2019). The assembly was checked for contamination and corrected using gEVAL ( Chow *et al.,* 2016) as described previously ( Howe *et al.,* 2021). Manual curation was performed using gEVAL, HiGlass ( Kerpedjiev *et al.,* 2018) and Pretext. The mitochondrial genome was assembled using MitoHiFi ( Uliano-Silva *et al.,* 2021), which performs annotation using MitoFinder ( Allio *et al.,* 2020). The genome was analysed and BUSCO scores generated within the BlobToolKit environment ( Challis *et al.,* 2020). Table 3 contains a list of all software tool versions used, where appropriate.

**Table 3.** Software tools used.

Software tool	Version	Source
Hifiasm	0.12	Cheng *et al.*, 2021
purge_dups	1.2.3	Guan *et al.*, 2020
SALSA2	2.2	Ghurye *et al.*, 2019
longranger align	2.2.2	https://support.10xgenomics.com/genome-exome/software/pipelines/latest/advanced/other-pipelines
freebayes	1.3.1-17-gaa2ace8	Garrison & Marth, 2012
MitoHiFi	1.0	Uliano-Silva *et al.*, 2021
gEVAL	N/A	Chow *et al.*, 2016
HiGlass	1.11.6	Kerpedjiev *et al.*, 2018
PretextView	0.1.x	https://github.com/wtsi-hpag/PretextView
BlobToolKit	3.0.5	Challis *et al.*, 2020

### Ethics/compliance issues

The materials that have contributed to this genome note have been supplied by a Darwin Tree of Life Partner. The submission of materials by a Darwin Tree of Life Partner is subject to the Darwin Tree of Life Project Sampling Code of Practice. By agreeing with and signing up to the Sampling Code of Practice, the Darwin Tree of Life Partner agrees they will meet the legal and ethical requirements and standards set out within this document in respect of all samples acquired for, and supplied to, the Darwin Tree of Life Project. Each transfer of samples is further undertaken according to a Research Collaboration Agreement or Material Transfer Agreement entered into by the Darwin Tree of Life Partner, Genome Research Limited (operating as the Wellcome Sanger Institute), and in some circumstances other Darwin Tree of Life collaborators.

## Data availability

European Nucleotide Archive: Tachina fera. Accession number PRJEB42946; https://identifiers.org/ena.embl/PRJEB42946.

The genome sequence is released openly for reuse. The *T. fera* genome sequencing initiative is part of the Darwin Tree of Life (DToL) project. All raw sequence data and the assembly have been deposited in INSDC databases. The genome will be annotated using the RNA-Seq data and presented through the Ensembl pipeline at the European Bioinformatics Institute. Raw data and assembly accession identifiers are reported in Table 1.

## Author information

Members of the University of Oxford and Wytham Woods Genome Acquisition Lab are listed here: https://doi.org/10.5281/zenodo.5746938.

Members of the Darwin Tree of Life Barcoding collective are listed here: https://doi.org/10.5281/zenodo.5744972.

Members of the Wellcome Sanger Institute Tree of Life programme are listed here: https://doi.org/10.5281/zenodo.6125027.

Members of Wellcome Sanger Institute Scientific Operations: DNA Pipelines collective are listed here: https://doi.org/10.5281/zenodo.5746904.

Members of the Tree of Life Core Informatics collective are listed here: https://doi.org/10.5281/zenodo.6125046.

Members of the Darwin Tree of Life Consortium are listed here: https://doi.org/10.5281/zenodo.5638618.
