# Supplementary material for: The genome sequence of Tachina fera (Linnaeus, 1761), a tachinid fly
Source: Wellcome Open Res. 2026 Jan 8;7:103. Originally published 2022 Mar 21. [Version 2] doi: 10.12688/wellcomeopenres.17760.2 (PMC12966796; doi:10.12688/wellcomeopenres.17760.2)
Supplement: Peer review history

# Reviewer report: Reviewer response for version 2

**DOI:** 10.21956/wellcomeopenres.27574.r147943

**Published:** 2026-03-06

**Authors:** Andrzej Grzywacz

**Affiliations:** 1 Department of Ecology and Biogeography, Nicolaus Copernicus University in Toruń, Toruń, Poland

The article is a Data Note on the chromosome-level genome assembly of *Tachina fera*. It provides a brief introduction, a summary of the obtained results, and descriptions of the laboratory protocols and bioinformatic analyses.

 The strong advantages of the paper are:

- The genome assembly of species representing mega-diverse dipteran family.
- The genome assembly is publicly available.
- The data generated during the study are publicly available.
- The data analysis is clearly described.

**Minor comments to improve the article:**

 Some additional information how the dataset can be used in the future would be welcomed (phylogenomics, studies on genomic adaptations to parasitism).

 Information regarding sample acquisition, DNA extraction, and sequencing could be provided in greater detail. For example, it would be useful to describe how the material was identified. It is always helpful to include a reference to the identification key used (information on the most recent species definition that the collector followed).

 In the sentence "The parasitic nature of Tachinid species...", please change "Tachinid" to lowercase ("tachinid"). Please check this usage throughout the manuscript.

 In the sentence "The chromosome-level genome assembly presented here is, to our knowledge, the first high-quality resource developed for a Tachinid...", this statement should be updated. Currently, several additional chromosome-level genome assemblies for Tachinidae are available. Since the first version of the manuscript was published, new genomes from Tachinidae have been assembled and made publicly available.

Are sufficient details of methods and materials provided to allow replication by others?

Yes

Is the rationale for creating the dataset(s) clearly described?

Partly

Are the datasets clearly presented in a useable and accessible format?

Yes

Are the protocols appropriate and is the work technically sound?

Yes

Reviewer Expertise:

Phylogenomics and evolutionary history of Diptera.

I confirm that I have read this submission and believe that I have an appropriate level of expertise to confirm that it is of an acceptable scientific standard.

# Reviewer report: Reviewer response for version 1

**DOI:** 10.21956/wellcomeopenres.19657.r51184

**Published:** 2022-08-08

**Authors:** Sheina Sim

**Affiliations:** 1 Daniel K. Inouye US Pacific Basin Agricultural Research Center, Tropical Crop and Commodity Protection Research Unit, USDA Agricultural Research Service, Hilo, HI, USA

The *Tachina fera *genome was sequenced and assembled to a chromosome length and represents the highest quality genome for a tachinid fly. The work described is appropriate for the goal and is well written, though missing some details.

**Major comments:**

- The details for some of the analysis methods were not defined. I see that there is a blobplot provided, and that requires additional analyses to determine the taxonomic identifications and depth of coverage for each fragment, but those details were not conveyed. 
- Please describe how the X chromosome was identified as those methods were not included in the manuscript. 
- The reason for conducting this genome assembly effort was not well articulated. What can you learn from this assembly? What will you use the assembly for? You describe the species as an under appreciated regulator of herbivory, how will the genome improve your understanding of the species?

**Minor comments:**

- The snail plot is divided into 100 (not 1000) size-ordered bins

Are sufficient details of methods and materials provided to allow replication by others?

Partly

Is the rationale for creating the dataset(s) clearly described?

Partly

Are the datasets clearly presented in a useable and accessible format?

Partly

Are the protocols appropriate and is the work technically sound?

Yes

Reviewer Expertise:

Insect genomics, population genetics, and bioinformatics.

I confirm that I have read this submission and believe that I have an appropriate level of expertise to confirm that it is of an acceptable scientific standard, however I have significant reservations, as outlined above.

# Reviewer report: Reviewer response for version 1

**DOI:** 10.21956/wellcomeopenres.19657.r49376

**Published:** 2022-03-22

**Authors:** Jaakko Pohjoismäki

**Affiliations:** 1 Department of Environmental and Biological Sciences, University of Eastern Finland, Joensuu, Finland

Thank you for the opportunity to review the data note manuscript by Will Nash *et al.* on the genome sequence of *Tachina fera* (Diptera: Tachinidae).

 The approach taken by DToL can be seen as a benchmark for many other genome sequencing initiatives for providing a state-of-the-art, chromosomally scaffolded reference genome for the species. It is great to see reference genomes from the magnificent and important group of true flies.

 I have only few minor suggestions to improve the content of the manuscript.

**Title:**

 The species author’s name needs to be in parentheses - *Tachina fera* (Linnaeus, 1761) [originally *Musca fera* Linnaeus, 1761]. For clarity, the insect order and family should be added to the title (Diptera, Tachinidae).

**Species taxonomy:**

 ibid for the author’s name.

**Background:**

 Very nice introduction. The abdomen is maybe more of orange than chestnut, although this can vary, and the females are often darker than the males. There is another very similar species in the UK (although apparently until now only on the Channel Islands), *Tachina magnicornis* (Zetterstedt, 1844). This is a bit problematic for the story as the females are difficult to tell apart with certainty. It is very unlikely that you would have happened to sample the rarer species, but you might need to make this reservation in the introduction.

 Figure 1 is quite poor when it comes to specimen details. For vouchering, I would recommend taking more detailed images. I would for example think (based on the shape of the abdomen and apparently narrow frons, although this is poorly visible) that this is a male specimen. Or can it be that the image is from the other specimen used for the RNA-seq?

**Genome sequence report:**

 Looks very good. To be sure of the specimen, you could check the sex by (most calyptrate flies follow XY-system of sex determination) by looking at the existence of the dominant male-determining factor in your sequence data (e.g. Vicoso & Bachtrog, 2015 ^1^). Was the X chromosome present as diploid or haploid sequence count?

 However, I see no presentation of the RNA-seq data? How many transcripts, what coverage etc?

**Sample acquisition & nucleic acids extraction:**

 Were the whole specimens destroyed in the DNA and RNA extractions or is there some reference tissue left? Where (and how) are these stored and how can they be located? If the reference specimens are still existing, describe all associated labels in detail. For later morphological analysis it would be great to preserve at least the abdomens and legs as a voucher. The voucher should be placed in a public collection. I am sure you have some established practice within DToL but it needs to be described here.

 Also: “The specimen was caught in […]” -> The specimen **s were** caught … (there was two).

**RNA extraction:**

 Was there no poly-A purification? How was the rRNA depleted before the sequencing?

**Suggestion for the future:** 

 Depending on your RNA-seq results, it might make sense to extract RNA from the head+thorax (especially when there are more than one specimen). The RNA yield is often poor from the abdomen (on average) due to high levels of digestive enzymes, mass of gut content, fat etc. Also the head+thorax could give a better overview of the gene expression.

Are sufficient details of methods and materials provided to allow replication by others?

Yes

Is the rationale for creating the dataset(s) clearly described?

Yes

Are the datasets clearly presented in a useable and accessible format?

Yes

Are the protocols appropriate and is the work technically sound?

Yes

Reviewer Expertise:

Molecular biology, genetics, population genetics, evolution, Diptera taxonomy

I confirm that I have read this submission and believe that I have an appropriate level of expertise to confirm that it is of an acceptable scientific standard.

## References

[1] : Numerous transitions of sex chromosomes in Diptera. *PLoS Biol* .2015;13(4) : 10.1371/journal.pbio.1002078 e1002078 10.1371/journal.pbio.1002078 25879221 PMC4400102
